# An observational study of drug utilization and associated outcomes among adult patients diagnosed with BRAF‐mutant advanced melanoma treated with first‐line anti‐PD‐1 monotherapies or BRAF/MEK inhibitors in a community‐based oncology setting

**DOI:** 10.1002/cam4.3312

**Published:** 2020-09-01

**Authors:** Charles L. Cowey, Marley Boyd, Kathleen M. Aguilar, April Beeks, Clemens Krepler, Emilie Scherrer

**Affiliations:** ^1^ Texas Oncology Baylor Charles A Sammons Cancer Center Dallas TX USA; ^2^ McKesson Life Sciences The Woodlands TX USA; ^3^ Merck & Co., Inc. Kenilworth NJ USA

**Keywords:** biomarkers, immunology, survival, target therapy

## Abstract

**Introduction:**

Anti‐PD‐1 monotherapies (aPD‐1) and BRAF/MEK inhibitors (BRAF/MEKi) changed the BRAF‐mutant advanced melanoma treatment landscape. This study aimed to improve the understanding of real‐world treatment patterns and optimal treatment sequence.

**Methods:**

This was a retrospective study of BRAF‐mutant advanced melanoma patients who initiated 1L aPD‐1 or BRAF/MEKi in the US Oncology Network between 1 January 2014 and 31 December 2017, followed through 31 December 2018. Patient and treatment characteristics were assessed descriptively, with Kaplan‐Meier methods used for time‐to‐event endpoints. As the primary analysis, overall survival (OS) and physician‐assessed progression‐free survival (rwPFS) were evaluated with Cox proportional hazard regression models and propensity score matching (n = 49).

**Results:**

A total of 224 patients were included (median age 61 years, 62.9% male, 89.7% white): 36.2% received aPD‐1 and 63.8% BRAF/MEKi. Median OS and rwPFS were longer among aPD‐1 vs BRAF/MEKi patients (OS: not reached vs 13.9 months, log‐rank *P* = .0169; rwPFS: 7.6 vs 6.5 months, log‐rank *P* = .0144). Receipt of aPD‐1 was associated with improved OS (HR = 0.602 vs BRAF/MEKi [95%CI 0.382‐0.949]; *P* = .0287). Among patients without an event within 6 months of 1L initiation, receipt of aPD‐1 was associated with a decreased risk of progression or death from 6 months onwards (HR = 0.228 [95%CI 0.106‐0.493]; *P* = .0002). This association was not observed among patients within 6 months of 1L initiation (HR = 1.146; 95% CI 0.755‐1.738). Results from the propensity score‐matched pairs were consistent with these trends.

**Conclusion:**

These results suggest a clinical benefit of 1L aPD‐1 compared to BRAF/MEKi after 6 months of treatment for BRAF‐mutant advanced melanoma. Future research should explore factors associated with early progression and their relationship with clinical outcomes.

## Graphical abstract

While both aPD‐1 and BRAF/MEKi are approved for BRAF‐mutant advanced melanoma, limited evidence exists comparing these therapies, particularly to understand the optimal treatment sequence. This study provides evidence of a clinical benefit of first‐line (1L) aPD‐1 compared to BRAF/MEKi after 6 months among patients with BRAF‐mutant advanced melanoma who received care in a large network of US community oncology clinics.

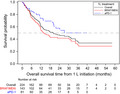

## 1 INTRODUCTION

In the United States, an estimated 96 480 people will be diagnosed with melanoma and 7230 will die from the disease in 2019.^1^ For the 84% of patients with malignant melanoma that are diagnosed with localized disease, the 5‐year survival rate is 98.7%; in contrast, the prognosis for patients diagnosed with metastatic melanoma is 24.8%.^2^

Novel targeted therapies and immunotherapies have changed the treatment landscape for advanced melanoma.^3^ Immunotherapies include ipilimumab, which targets cytotoxic T lymphocyte‐associated protein 4, as well as pembrolizumab and nivolumab, which target programmed death protein‐1 (PD‐1). These agents have demonstrated favorable trial results.^4^

Five‐year data from KEYNOTE‐006 showed superior outcomes associated with pembrolizumab compared with ipilimumab among previously treated advanced melanoma patients.^5^ Across the KEYNOTE‐006 population, median overall survival (OS) was 32.7 months (95% confidence interval [CI] 24.5‐41.6) vs 15.9 months (95%CI 13.3‐22.0), respectively; median progression‐free survival (PFS) was 8.4 months (95%CI 6.6‐11.3) vs 3.4 months (95%CI 2.9‐4.2), respectively. Among a subset of treatment‐naïve patients, median PFS and OS were also longer for those who received pembrolizumab compared to ipilimumab (OS: 11.6 months [95%CI 8.2‐16.4] vs 3.7 months [95%CI 2.8‐4.3], hazard ratio [HR]0.54 [95%CI 0.44‐0.67], *P* < .0001; PFS: 38.7 months [95%CI 27.3‐50.7] vs 17.1 months [95%CI 13.8‐26.2], HR = 0.73 [95%CI 0.57‐0.92], *P* = .0036).

Similarly, for CheckMate 067, median OS was longer among treatment‐naïve advanced melanoma nivolumab monotherapy than those who received ipilimumab monotherapy (36.9 months [95%CI 28.2‐58.7] vs 19.9 months [95%CI 16.8‐24.6], respectively; HR 0.63 [95%CI 0.52‐0.76]; *P* < .001)).^4^ In this trial, patients who received nivolumab/ipilimumab also demonstrated a trend toward improved OS compared to those who received ipilimumab monotherapy.

Targeted therapies are indicated for the approximately 50% of melanoma patients with a BRAF V600 mutation.^6^, ^7^ For these patients, BRAF/MEK inhibitors (BRAF/MEKi), including dabrafenib/trametinib, vemurafenib/cobimetinib, and encorafenib/binimetinib, delay resistance associated with BRAF inhibition alone.^3^, ^8^, ^9^ These agents demonstrated favorable results in trials of treatment‐naïve patients with unresectable BRAF‐mutant melanoma.^8^, ^9^ According to 3‐year data from COMBI‐d, median OS was longer among dabrafenib/trametinib‐treated patients: 25.1 months (95%CI 19.2‐not reached [NR]) vs 18.7 months (95%CI 15.2‐23.7) with dabrafenib monotherapy, and median PFS was 11.0 months (95%CI 8.0‐13.9) vs 8.8 months (95%CI 5.9‐9.3), respectively.^8^ Five‐year survival and PFS rates were 28% and 13%, respectively.^9^ In COMBI‐v, dabrafenib/trametinib had superior median OS vs vemurafenib monotherapy (NR vs 17.2 months, respectively) and median PFS (11.4 vs 7.3 months, respectively).^10^

Anti‐PD‐1 monotherapies (aPD‐1) are also associated with improved outcomes among patients with BRAF‐mutant melanoma.^10^, ^11^, ^12^, ^13^, ^14^, ^15^, ^16^, ^17^ KEYNOTE‐006 reported that, among previously BRAF‐mutant advanced melanoma patients, median OS was 20.4 months (95%CI 12.8‐35.6) among those who received pembrolizumab and 11.9 months (95%CI 6.0‐17.8) for those who received ipilimumab (HR = 0.71 [95%CI 0.46‐1.08], *P* = .054).^5^ Among treatment‐naïve BRAF‐mutant patients, median OS was not reached (95%CI 36.1‐NR) for those who received pembrolizumab and 26.2 months (95%CI 16.0‐NR) for those who received ipilimumab (HR = 0.70 [95%CI 0.44‐1.11} *P* = .065).

Moser et al (2019) retrospectively assessed electronic health record (EHR) data of patients with BRAF‐mutant melanoma who received first‐line (1L) treatment with aPD‐1, BRAF/MEKi, or nivolumab/ipilimumab.^18^ Median OS was longest among those who received nivolumab/ipilimumab and aPD‐1, compared to those who received BRAF/MEKi (NR [interquartile range (IQR) 8.7‐NR] and 39.5 months [IQR 8.7‐NR] vs 13.2 months [IQR 5.2‐41.4]).

While both BRAF/MEKi^3^, ^8^, ^9^ and aPD‐1^10^, ^12^ have the US Food and Drug Administration (FDA) approval for patients with advanced BRAF‐mutant melanoma, limited evidence exists comparing those treatment options. Additionally, data are sparse concerning optimal strategies for treatment sequencing in advanced melanoma, especially for BRAF‐mutant disease. Most studies have investigated outcomes associated with a single line of therapy, without consideration to how patients’ prior and subsequent treatments may have impacted clinical outcomes. Long‐term OS results may not accurately reflect efficacy associated specifically with 1L therapies as opposed to all therapies combined. While studies of treatment sequencing are in progress, these data are not yet available to support clinical decision‐making. The aim of this study was therefore to assess treatment patterns and outcomes among patients with BRAF‐mutant advanced melanoma who initiated 1L treatment with aPD‐1 or BRAF/MEKi in a large network of community oncology clinics. Patients were followed as they advanced through their treatment sequences.

## 2 MATERIALS AND METHODS

### 2.1 Study design and data sources

This was a retrospective cohort study of the US adult patients with BRAF‐mutant advanced melanoma who initiated 1L treatment with aPD‐1 or BRAF/MEKi combination therapy between 01 January 2014 and 31 December 2017 from practices in the US Oncology Network (USON). Patients were followed until their last record, death date, or end of the study period (31 December 2018), whichever occurred first. This was an extension of previous studies of advanced melanoma patients who initiated 1L treatment within the USON.^19^, ^20^, ^21^

The combination of nivolumab/ipilimumab was granted accelerated approval for advanced melanoma patients with BRAF mutations in September 2015 and, regardless of BRAF status, in January 2016.^22^ Given the period of observation, the decision was made to focus this study on patients who received 1L aPD‐1 or BRAF/MEKi due to the limited number of evaluable patients who received nivolumab/ipilimumab during the time period of analysis.^19^

The USON is affiliated with approximately 1400 physicians in more than 470 sites of care across 25 states in the US, representing approximately 12% of the US patients newly diagnosed with cancer.^23^ Data were obtained via programmatic database extraction from the EHR of the USON, iKnowMed. iKnowMed is an oncology‐specific EHR system that captures outpatient practice encounter history for patients receiving treatment within the USON, including laboratory tests, diagnosis, administration of infused therapies, prescription of oral therapies, staging, comorbidities, and performance status. This product has been implemented across the USON. Data were first extracted from structured EHR fields, with chart review performed to capture and/or verify other information of interest, including physician‐assessed response, patients’ treatment histories, and dates of diagnoses. Supplemental vital status information was provided by the Social Security Administration's Limited Access Death Master File.

Eligible patients were at least 18 years of age at advanced melanoma diagnosis and had at least two visits within the USON or a record of death during the study observation period. Patients were excluded if they were enrolled in clinical trials or had another primary cancer diagnosis during the study period (with the exception of basal cell carcinoma, squamous cell carcinoma, bladder carcinoma in situ, and cervical carcinoma in situ).

The study was reviewed and granted an exception and waiver of consent by the US Oncology, Inc Institutional Review Board.

### 2.2 Statistical analysis

Patient demographic, clinical, and treatment characteristics were assessed descriptively, with the Kaplan‐Meier method used to assess time‐to‐event endpoints, including OS, physician‐assessed PFS (rwPFS), time to treatment discontinuation (TTD), time to next treatment (TTNT), and duration of physician‐assessed response (DOR).

OS was defined as the duration from 1L treatment initiation until death from any cause, with censoring of patients who did not die by the end of the study period. TTD was defined as the duration from 1L treatment initiation to discontinuation for any reason, with censoring of patients who did not discontinue 1L treatment by the end of the study period. Date of discontinuation was captured through chart review as the stop date explicitly documented by a provider (for oral therapies), last administration date (for infused therapies), date of hospice admission, or death date. TTNT was defined as the duration from 1L treatment initiation to the start of a new treatment, with censoring of patients who did not initiate a new treatment by the end of the study period. rwPFS was defined as the duration from 1L treatment initiation until either physician‐documented disease progression or death, with censoring of patients who did not die or experience physician‐documented disease progression by the end of the study period. DOR was defined as the duration from first documented response to 1L treatment initiation until date of first documented disease progression, with censoring of patients who did not experience response followed by disease progression by the end of the study period. For all time‐to‐event analyses, patients who did not experience the event were censored at the end of the study period, last visit date recorded, or death, whichever came first.

The primary objective of this study was to use multivariable Cox proportional hazard regression models and propensity score matching to adjust for underlying differences between the aPD‐1 and BRAF/MEKi cohorts that may have influenced OS and rwPFS. First, univariate and multivariable Cox proportional hazard regression models were used to identify independent risk factors for OS and rwPFS. To construct these models, all baseline and 1L treatment covariates were included in univariate models, and a stepwise selection approach was used to identify covariates for the multivariable models, with *P* ≤ .25 for entry and ≤.15 for retention.

Next, a propensity score matching technique was used to reduce confounding of variables that may have influenced outcomes. To balance the cohorts for this analysis, patients initiating BRAF/MEKi prior to 01 September 2014 were removed to align with the first FDA approval date of pembrolizumab for patients with melanoma, which was an accelerated approval for patients no longer responding to other therapies.^24^ Potential confounders included in the model were the following: age at 1L initiation, practice region, presence of other metastases, albumin result at 1L initiation, presence of bone metastases, radiation prior to 1L initiation, Deyo‐adapted Charlson score, presence of brain metastases, smoking history, Eastern Cooperative Oncology Group (ECOG) at 1L initiation, presence of liver metastases, stage at initial diagnosis, gender, presence of lung metastases, and surgical resection prior to 1L initiation.

A combination of univariate logistic regression results, consideration of clinically relevant variables, and stepwise selection was used to determine variables for inclusion into the multivariable logistic regression model. The multivariable logistic regression model using the chosen covariates was used to generate the propensity score. Once the score was generated, a greedy matching algorithm was used to pair up patients from both treatment cohorts. Attrition was expected and the remaining cohorts were verified to be well balanced between clinically relevant variables that may have otherwise confounded outcomes.

An alpha level of .05 was the primary criterion for statistical significance. Analyses were conducted using SAS^®^ 9.4 (SAS Institute Inc).

## 3 RESULTS

### 3.1 Demographic and clinical characteristics

In total, 224 patients met eligibility criteria (Figure S1). Eighty‐one (36.2%) received 1L aPD‐1 (67.9% pembrolizumab and 32.1% nivolumab), while 143 (63.8%) received 1L BRAF/MEKi (90.2% dabrafenib/trametinib and 9.8% cobimetinib/vemurafenib; Table 1). The median follow‐up durations of aPD‐1 and BRAF/MEKi patients were 11.3 (range, 0.4‐41.9) and 11.5 (range, 0.2‐58.2) months, respectively.

**TABLE 1:** Demographic, clinical characteristics, and treatment patterns for patients with BRAF‐mutant advanced melanoma

	Overall (n = 224)	aPD‐1 (n = 81)	BRAF/MEKi (n = 143)
Median age at 1L initiation, years (range)	61 (26, 90+)	62 (26, 90+)	61 (28, 90+)
Race, n (%)			
White	201 (89.7)	69 (85.2)	132 (92.3)
Unknown	18 (8.0)	7 (8.6)	11 (7.7)
Other	5 (2.2)	5 (6.2)	0 (0.0)
Male sex, n (%)	141 (62.9)	50 (61.7)	91 (63.6)
Median follow‐up time from 1L initiation, months (range)	11.5 (0.2, 58.2)	11.3 (0.4, 41.9)	11.5 (0.2, 58.2)
ECOG performance status at 1L initiation, n (%)			
0‐1	142 (63.4)	61 (75.3)	81 (56.6)
2+	35 (15.6)	4 (4.9)	31 (21.7)
Not documented	47 (21.0)	16 (19.8)	31 (21.7)
Stage at diagnosis, n (%)			
Stage I/II	21 (9.4)	6 (7.4)	15 (10.5)
Stage III/IV	167 (74.6)	51 (63.0)	116 (81.1)
Not documented	36 (16.1)	24 (29.6)	12 (8.4)
PD‐L1 status, n (%)			
Positive	9 (4.0)	6 (7.4)	3 (2.1)
Negative	16 (7.1)	9 (11.1)	7 (4.9)
Not documented	199 (88.8)	66 (81.5)	133 (93.0)
LDH status at 1L initiation^a^, n (%)			
Normal	93 (41.5)	41 (50.6)	52 (36.4)
Elevated	53 (23.7)	15 (18.5)	38 (26.6)
Not documented	78 (34.8)	25 (30.9)	53 (37.1)
Sites of metastases at 1L initiation, n (%)			
Other	150 (67.0)	45 (55.6)	105 (73.4)
Lung	101 (45.1)	23 (28.4)	78 (54.5)
Brain	70 (31.3)	17 (21.0)	53 (37.1)
Bone	59 (26.3)	17 (21.0)	42 (29.4)
Liver	51 (22.8)	7 (8.6)	44 (30.8)
Metastatic status at 1L initiation, n (%)			
M0	1 (0.4)	0 (0.0)	1 (0.7)
M1a	19 (8.5)	9 (11.1)	10 (7.0)
M1b	26 (11.6)	9 (11.1)	17 (11.9)
M1c	145 (64.7)	44 (54.3)	101 (70.6)
Mx	33 (14.7)	19 (23.5)	14 (9.8)
Median time from advanced melanoma diagnosis to 1L initiation, months (range)	1.1 (0.0, 43.2)	1.4 (0.0, 43.2)	1.0 (0.0, 27.1)
Median duration of 1L therapy, months (range)	5.0 (0.0, 57.4)	3.7 (0.0, 41.9)	5.4 (0.0, 57.4)
1L regimen, n (%)			
Dabrafenib/trametinib	129 (57.6)	0 (0.0)	129 (90.2)
Pembrolizumab	55 (24.6)	55 (67.9)	0 (0.0)
Nivolumab	26 (11.6)	26 (32.1)	0 (0.0)
Cobimetinib/vemurafenib	14 (6.3)	0 (0.0)	14 (9.8)
Radiation prior to 1L initiation, n (%)	85 (37.9)	30 (37.0)	55 (38.5)
Prior radiation and brain metastases at 1L initiation, n (%)	48 (21.4)	13 (16.0)	35 (24.5)
Surgical resection prior to 1L initiation, n (%)	155 (69.2)	61 (75.3)	94 (65.7)
Patients who discontinued 1L treatment, n (%)	196 (87.5)	62 (76.5)	134 (93.7)
Reasons for 1L treatment discontinuation, n (%)			
Disease progression	79 (40.3)	28 (45.2)	51 (38.1)
Other	29 (14.8)	10 (16.1)	19 (14.2)
Treatment‐related toxicities	26 (13.3)	7 (11.3)	19 (14.2)
Death	16 (8.2)	2 (3.2)	14 (10.4)
Patient choice	6 (3.1)	4 (6.5)	2 (1.5)
Decline in ECOG	6 (3.1)	1 (1.6)	5 (3.7)
Unknown	54 (27.6)	14 (22.6)	40 (29.9)
Patients who advanced to 2L, n (%)	119 (53.1)	35 (43.2)	84 (58.7)
Median duration of 2L therapy, months (range)	2.1 (0.0, 34.6)	2.8 (0.0, 34.6)	2.1 (0.0, 25.1)
2L regimen, n (% of 2L initiators)			
Pembrolizumab	31 (26.1)	2 (5.7)	29 (34.5)
Nivolumab/ipilimumab	26 (21.8)	3 (8.6)	23 (27.4)
Dabrafenib/trametinib	20 (16.8)	20 (57.1)	0 (0.0)
Nivolumab	16 (13.4)	0 (0.0)	16 (19.0)
Ipilimumab	9 (7.6)	3 (8.6)	6 (7.1)
Other^b^	17 (14.3)	7 (20.0)	10 (11.9)
Reasons for 2L treatment initiation, n (% of 2L initiators)			
Progression on prior therapy	80 (67.2)	26 (74.3)	54 (64.3)
Other	16 (13.4)	5 (14.3)	11 (13.1)
Toxicity on prior therapy	10 (8.4)	4 (11.4)	6 (7.1)
Unknown	15 (12.6)	1 (2.9)	14 (16.7)
Patients who discontinued 2L treatment, n (% of 2L initiators)	100 (84.0)	26 (74.3)	74 (88.1)
Reasons for 2L treatment discontinuation, n (% of 2L discontinuations)			
Disease progression	21 (21.0)	4 (15.4)	17 (23.0)
Treatment‐related toxicities	14 (14.0)	8 (30.8)	6 (8.1)
Patients who advanced to 3L, n (% of 2L discontinuations)	40 (17.9)	5 (6.2)	35 (24.5)

Abbreviations: 1L, first‐line; 2L, second‐line; 3L, third‐line; aPD‐1, anti‐PD‐1 monotherapies; BRAF/MEKi, BRAF/MEK inhibitors; ECOG, Eastern Cooperative Oncology Group; LDH, lactate dehydrogenase; PD‐L1, programmed death ligand‐1.

^a^ LDH thresholds were recorded in the EHR based on individual laboratory specifications; standard reference ranges were not used.

^b^ Other 2L treatments were received by fewer than five patients in each cohort.

Median age at diagnosis was 61 years, 62.9% were male, 89.7% were white, 48.2% had Stage IV disease, and 63.4% had an ECOG performance status 0‐1 (Table 1). A higher proportion of aPD‐1 patients had ECOG 0‐1 performance status compared with the BRAF/MEKi group. Most patients in both treatment groups had M1c metastatic status, with higher proportions of the 1L BRAF/MEKi group having documented lung, brain, and liver metastases. Lactate dehydrogenase (LDH) status as 1L treatment initiation was recorded as being “elevated” in the EHR among 18.5% of aPD‐1 patients and 26.6% of BRAF/MEKi patients.

KIT, NRAS, and PD‐L1 status were not documented for the majority of patients in both treatment groups (Table 1). A higher proportion of the 1L BRAF/MEKi group lacked documentation of PD‐L1 status (93.0% vs 81.5%).

### 3.2 Treatment characteristics

Among aPD‐1 patients, 37.0% had prior radiation and 63.0% had prior surgery, with 38.5% and 61.5%, respectively, in the BRAF/MEKi group (Table 1). In total, 21.4% (n = 48) of patients had both prior radiation and brain metastases at 1L treatment initiation (24.5% [n = 35] of BRAF/MEKi and 16.0% [n = 13] of aPD‐1 patients). Median time from diagnosis to 1L treatment initiation was longer among the aPD‐1 group vs the BRAF/MEKi group: 1.4 (range, 0.0‐43.2) vs 1.0 (range, 0.0‐27.1) months (*P* = .0046).

Across the study population, 87.5% discontinued their 1L treatment; a higher proportion of patients who received 1L BRAF/MEKi discontinued compared with those who received 1L aPD‐1 (93.7% vs 76.5%, respectively, *P* = .0002; Table 1). The top two discontinuation reasons in the aPD‐1 group were disease progression (45.2%) and treatment‐related toxicities (11.3%). Similarly, the two most common discontinuation reasons in the BRAF/MEKi group were disease progression (38.1%) and treatment‐related toxicities (14.2%). Discontinuation of 1L due to death occurred among 3.2% of the aPD‐1 group and 10.4% of the BRAF/MEKi group.

Among those who discontinued, a higher proportion of the BRAF/MEKi group advanced to 2L treatment compared with the aPD‐1 group (58.7% vs 43.2%, respectively; Table 1). The most common 2L treatments among aPD‐1 patients who received 2L treatment were dabrafenib/trametinib (57.1%), nivolumab/ipilimumab (8.6%), and ipilimumab monotherapy (8.6%); and in the BRAF/MEKi group, they were pembrolizumab monotherapy (34.5%), nivolumab/ipilimumab (27.4%), and nivolumab monotherapy (19.0%; among those who received 2L treatments).

In total, 6.2% of the aPD‐1 group and 24.5% of the BRAF/MEKi group advanced to 3L (Table 1).

### 3.3 Clinical outcomes

**FIGURE 1:**
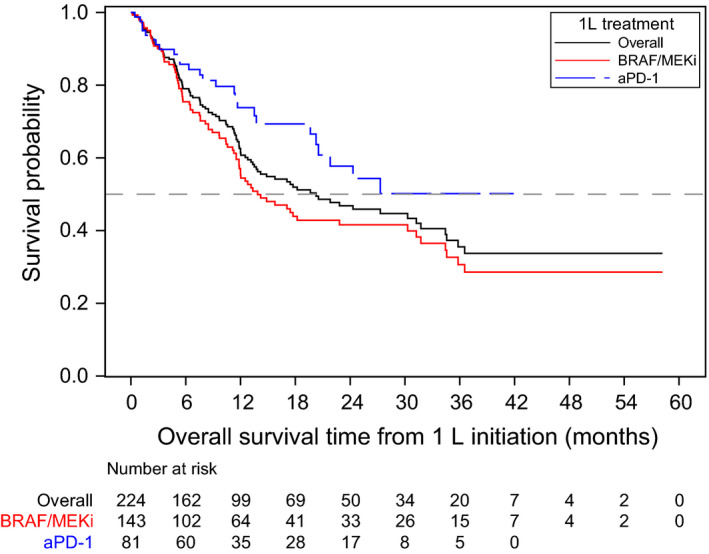
Kaplan‐Meier curve for overall survival. Abbreviations: 1L, first‐line; aPD‐1, anti‐PD‐1 monotherapy; BRAF/MEKi, BRAF/MEK combination therapy

Median OS from 1L treatment initiation was significantly longer among the aPD‐1 group compared with the BRAF/MEKi group (NR; [95%CI 20.3‐NR] vs 13.9 months [95%CI 11.8‐30.3], respectively; log‐rank *P* = .0169; Table 2, Figure 1). At 12 and 24 months, the survival rates were 73.8% and 57.7% for patients who received aPD‐1, respectively (data not shown). The 12‐ and 24‐month survival rates for patients who received BRAF/MEKi were 55.3% and 41.6%, respectively.

**TABLE 2:** Summary of Kaplan‐Meier time‐to‐event analyses

Outcome (in months; 95%CI)	Overall (n = 224)	aPD‐1 (n = 81)	BRAF/MEKi (n = 143)	Log‐rank *P*‐value
OS	20.3 (13.7, 31.7)	NR (20.3, NR)	13.9 (11.8, 30.3)	.0169
TTD	5.1 (4.5, 6.0)	4.4 (3.2, 6.0)	5.5 (4.9, 6.3)	.7776
TTNT	6.6 (5.7 ,8.0)	7.3 (5.2, 15.2)	6.5 (5.6, 7.8)	.0111
rwPFS	6.7 (5.7, 8.1)	7.6 (4.9, 19.6)	6.5 (5.6, 8.1)	.0144
DOR	11.8 (6.3, 23.2)	NR (15.9, NR)	6.6 (3.5, 11.8)	.0049

Abbreviations: aPD‐1, anti‐PD‐1 monotherapies; BRAF/MEKi, BRAF/MEK inhibitors; CI, confidence interval; DOR, duration of response; NR; not reported; OS, overall survival; rwPFS, physician‐assessed progression‐free survival; TTD, time to treatment discontinuation; TTNT, time to next treatment.

**FIGURE 2:**
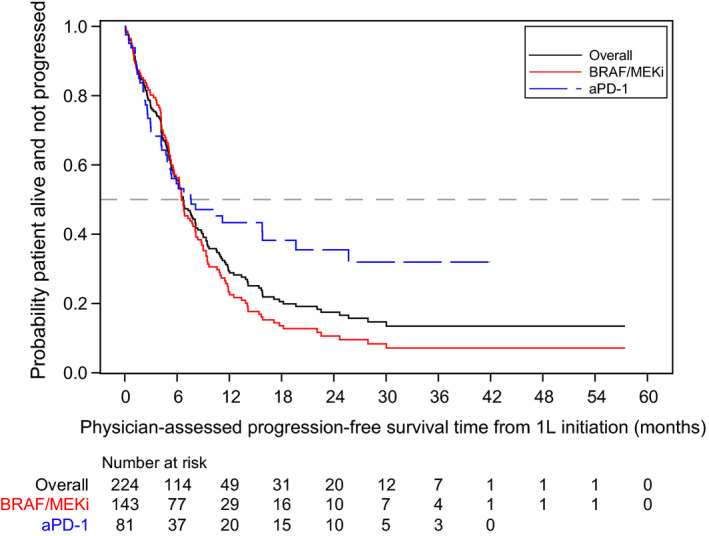
Kaplan‐Meier curve for physician‐assessed progression‐free survival. Abbreviations: 1L, first‐line; aPD‐1, anti‐PD‐1 monotherapy; BRAF/MEKi, BRAF/MEK combination therapy

Median rwPFS from 1L treatment initiation was higher among the aPD‐1 group compared with the BRAF/MEKi group (7.6 [95%CI 4.9‐19.6] vs 6.5 months [95%CI 5.6‐8.1], respectively; log‐rank *P* = .0144; Table 2, Figure 2).

**FIGURE 3:**
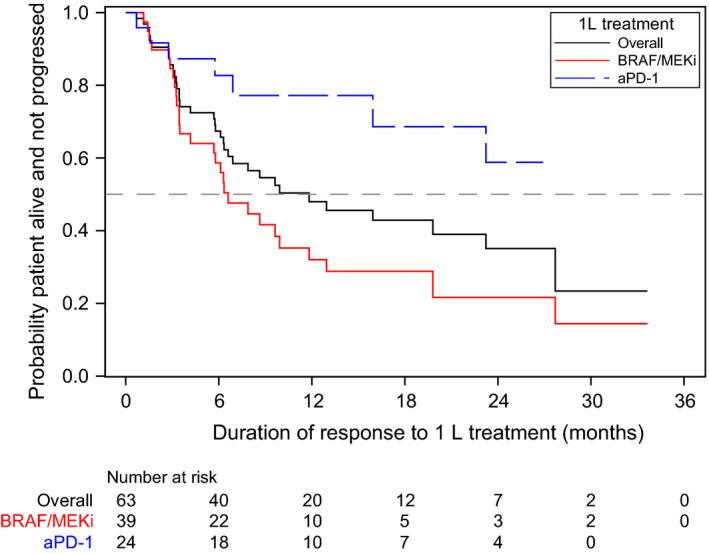
Kaplan‐Meier curve for duration of response to 1L treatment. Abbreviations: 1L, first‐line; aPD‐1, anti‐PD‐1 monotherapy; BRAF/MEKi, BRAF/MEK combination therapy

Median DOR from 1L initiation was not reached (95%CI 15.9‐NR) among the aPD‐1 group and 6.6 months (95%CI 3.5‐11.8) among the BRAF/MEKi group (log‐rank *P* = .0049; Table 2, Figure 3).

**FIGURE 4:**
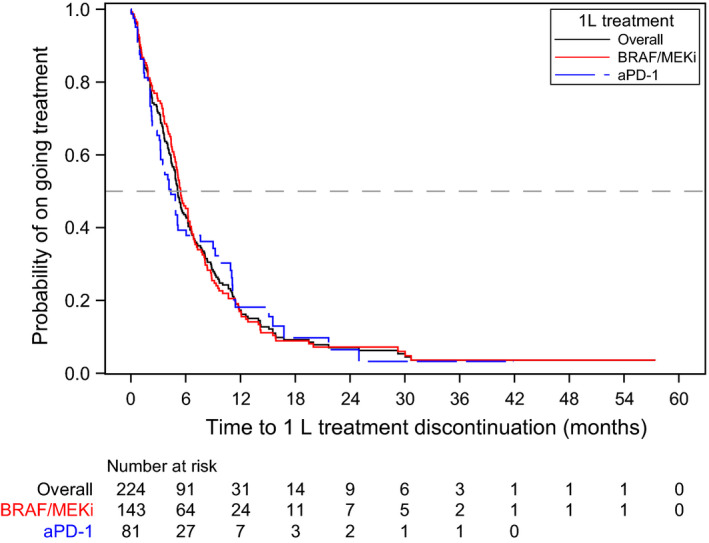
Kaplan‐Meier curve for time to treatment discontinuation. Abbreviations: 1L, first‐line; 2L; second‐line; aPD‐1, anti‐PD‐1 monotherapy; BRAF/MEKi, BRAF/MEK combination therapy

Median TTD from 1L initiation was not significantly different between the treatment groups, although there was a numerically longer duration among the BRAF/MEKi group (5.5 [95%CI 4.9‐6.3] vs 4.4 months [95%CI 3.2‐6.0], respectively; log‐rank *P* = .7776; Table 2, Figure 4).

**FIGURE 5:**
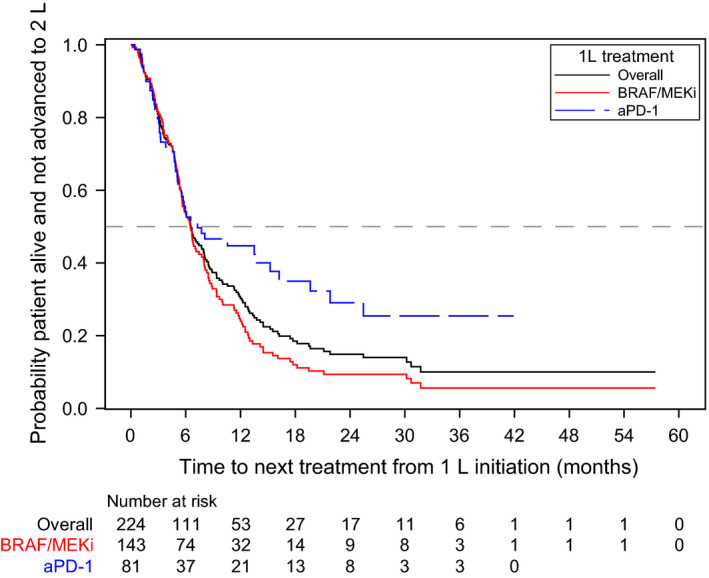
Kaplan‐Meier curve for time to next treatment. Abbreviations: 1L, first‐line; 2L; second‐line; aPD‐1, anti‐PD‐1 monotherapy; BRAF/MEKi, BRAF/MEK combination therapy

Median TTNT from 1L initiation was significantly longer among the aPD‐1 group compared with the BRAF/MEKi group (7.3 months [95%CI 5.2‐15.2] vs 6.5 months [95% 5.6‐7.8], respectively; log‐rank *P* = .0111; Table 2, Figure 5).

Univariate results are presented in Table 3. Based on the multivariable Cox regression results, age at 1L treatment initiation (HR = 1.044 with each year increase [95%CI 1.027‐1.063]; *P* < .0001), advanced stage at diagnosis (HR = 2.053 vs early stage [95%CI 1.025‐4.111; *P* = .0423), presence of brain metastases (HR = 2.192 [95%CI 1.452‐3.309]; *P* = .0002), and elevated LDH (HR = 2.146 [95%CI 1.327‐3.471]; *P* = .0019) were significantly associated with an increased risk of death (Table 4). In contrast, receipt of aPD‐1 (HR = 0.602 vs BRAF/MEK combination treatment [95%CI 0.382‐0.949]; *P* = .0287) was significantly associated with improved OS.

**TABLE 3:** Univariate Cox regression models on overall survival and physician‐assessed progression‐free survival from 1L treatment initiation

Covariate	Level	Total	Event (censored)	HR (95%CI)	*P*‐value
OS					
Age at 1L initiation	Per year increase	224	106 (118)	1.027 (1.012, 1.044)	.0006
Age group	<=65 (reference)	140	57 (83)	—	—
	>65	84	49 (35)	1.795 (1.224, 2.631)	.0027
Sex	Female (reference)	83	37 (46)	—	—
	Male	141	69 (72)	1.204 (0.807, 1.796)	.3635
ECOG at 1L initiation	0‐1 (reference)	142	66 (76)	—	—
	2+	35	22 (13)	1.922 (1.185, 3.117)	.0081
	Not documented	47	18 (29)	0.678 (0.402, 1.145)	.1463
BMI at 1L initiation	Normal (reference)	60	33 (27)	—	—
	Underweight	4	2 (2)	1.251 (0.3, 5.229)	.7585
	Overweight	80	41 (39)	0.855 (0.54, 1.352)	.5028
	Obese	73	28 (45)	0.558 (0.337, 0.925)	.0236
	Not documented	7	2 (5)	0.663 (0.158, 2.772)	.5731
Smoking status	Never (reference)	107	49 (58)	—	—
	Current	34	18 (16)	1.277 (0.743, 2.196)	.3756
	Former	79	37 (42)	1.219 (0.795, 1.869)	.3639
	Not documented	4	2 (2)	1.866 (0.451, 7.72)	.3894
Stage at diagnosis	Stage I/II (reference)	21	9 (12)	—	—
	Stage III/IV	167	91 (76)	1.824 (0.914, 3.64)	.0881
	Not documented	36	6 (30)	0.701 (0.248, 1.98)	.5031
Deyo‐adapted Charlson Score	0 (reference)	91	35 (56)	—	—
	1 or 2	105	57 (48)	1.533 (1.005, 2.337)	.0473
	3+	28	14 (14)	1.458 (0.784, 2.712)	.234
Albumin result	Normal (reference)	136	60 (76)	—	—
	Low	62	38 (24)	1.849 (1.229, 2.783)	.0032
	Not documented	26	8 (18)	0.531 (0.253, 1.114)	.0942
Bilirubin result	Normal (reference)	187	92 (95)	—	—
	Low	3	2 (1)	1.396 (0.343, 5.683)	.641
	Elevated	8	4 (4)	0.961 (0.352, 2.625)	.9379
	Not documented	26	8 (18)	0.442 (0.214, 0.915)	.0277
AST result	Normal (reference)	178	85 (93)	—	—
	Elevated	19	12 (7)	1.707 (0.929, 3.138)	.0849
	Not documented	27	9 (18)	0.52 (0.261, 1.038)	.0639
ALT result	Normal (reference)	133	70 (63)	—	—
	Low	2	1 (1)	1.072 (0.149, 7.738)	.9448
	Elevated	25	10 (15)	0.751 (0.387, 1.458)	.3978
	Not documented	64	25 (39)	0.647 (0.409, 1.021)	.0617
LDH result	Normal (reference)	93	41 (52)	—	—
	Elevated	53	32 (21)	1.687 (1.062, 2.681)	.0269
	Not documented	78	33 (45)	0.986 (0.622, 1.562)	.9517
Presence of bone metastases	No (reference)	165	71 (94)	—	—
	Yes	59	35 (24)	1.651 (1.101, 2.477)	.0153
Presence of brain metastases	No (reference)	154	64 (90)	—	—
	Yes	70	42 (28)	1.754 (1.186, 2.594)	.0049
Presence of liver metastases	No (reference)	173	72 (101)	—	—
	Yes	51	34 (17)	2.243 (1.488, 3.381)	.0001
Presence of lung metastases	No (reference)	123	45 (78)	—	—
	Yes	101	61 (40)	1.702 (1.157, 2.504)	.0069
Presence of other metastases	No (reference)	74	34 (40)	—	—
	Yes	150	72 (78)	0.869 (0.577, 1.309)	.502
Metastatic status at 1L initiation	M1c (reference)	145	79 (66)	—	—
	M0	1	1 (0)	1.594 (0.221, 11.5)	.6435
	M1a	19	3 (16)	0.158 (0.05, 0.504)	.0018
	M1b	26	14 (12)	0.715 (0.403, 1.267)	.2504
	Mx	33	9 (24)	0.426 (0.213, 0.852)	.0158
Radiation prior to 1L initiation	No (reference)	139	64 (75)	—	—
	Yes	85	42 (43)	1.192 (0.806, 1.762)	.3795
Surgical resection prior to 1L initiation	No (reference)	69	33 (36)	—	—
	Yes	155	73 (82)	0.828 (0.549, 1.25)	.37
1L treatment	BRAF/MEKi (reference)	143	80 (63)	—	—
	aPD‐1	81	26 (55)	0.586 (0.376, 0.914)	.0183
rwPFS					
Age at 1L initiation	Per year increase	224	168 (56)	1.007 (0.995, 1.019)	.2691
Age group	<=65 (reference)	140	104 (36)	—	—
	>65	84	64 (20)	1.269 (0.929, 1.734)	.1349
Sex	Female (reference)	83	63 (20)	—	—
	Male	141	105 (36)	1.138 (0.832, 1.555)	.4193
ECOG at 1L initiation	0‐1 (reference)	142	99 (43)	—	—
	2+	35	31 (4)	1.711 (1.141, 2.564)	.0093
	Not documented	47	38 (9)	1.138 (0.782, 1.655)	.4994
BMI at 1L initiation	Normal (reference)	60	46 (14)	—	—
	Underweight	4	2 (2)	0.579 (0.14, 2.387)	.4496
	Overweight	80	64 (16)	0.931 (0.637, 1.36)	.7103
	Obese	73	52 (21)	0.741 (0.498, 1.102)	.1386
	Not documented	7	4 (3)	0.84 (0.301, 2.343)	.7387
Smoking status	Never (reference)	107	80 (27)	—	—
	Current	34	26 (8)	1.129 (0.725, 1.758)	.5924
	Former	79	59 (20)	1.083 (0.774, 1.516)	.642
	Not documented	4	3 (1)	1.815 (0.572, 5.759)	.3116
Stage at diagnosis	Stage I/II (reference)	21	17 (4)	—	—
	Stage III/IV	167	135 (32)	1.264 (0.763, 2.096)	.3632
	Not documented	36	16 (20)	0.837 (0.421, 1.662)	.6114
Deyo‐adapted Charlson Score	0 (reference)	91	68 (23)	—	—
	1 or 2	105	80 (25)	1.017 (0.735, 1.408)	.917
	3+	28	20 (8)	1 (0.607, 1.648)	.9994
Albumin result	Normal (reference)	136	97 (39)	—	—
	Low	62	51 (11)	1.483 (1.054, 2.087)	.0235
	Not documented	26	20 (6)	1.028 (0.635, 1.666)	.9091
Bilirubin result	Normal (reference)	187	139 (48)	—	—
	Low	3	3 (0)	1.412 (0.449, 4.441)	.5554
	Elevated	8	6 (2)	0.959 (0.423, 2.173)	.9195
	Not documented	26	20 (6)	0.92 (0.575, 1.472)	.727
AST result	Normal (reference)	178	131 (47)	—	—
	Elevated	19	16 (3)	1.768 (1.051, 2.973)	.0318
	Not documented	27	21 (6)	1.007 (0.634, 1.598)	.977
ALT result	Normal (reference)	133	98 (35)	—	—
	Low	2	1 (1)	0.552 (0.077, 3.964)	.5549
	Elevated	25	18 (7)	1.083 (0.654, 1.791)	.7573
	Not documented	64	51 (13)	1.155 (0.823, 1.621)	.4044
LDH result	Elevated (reference)	53	43 (10)	1.083 (0.654, 1.791)	.7573
	Not documented	78	60 (18)	1.155 (0.823, 1.621)	.4044
Presence of bone metastases	No (reference)	165	121 (44)	—	—
	Yes	59	47 (12)	1.294 (0.924, 1.814)	.1339
Presence of brain metastases	No (reference)	154	105 (49)	—	—
	Yes	70	63 (7)	1.572 (1.148, 2.153)	.0048
Presence of liver metastases	No (reference)	173	120 (53)	—	—
	Yes	51	48 (3)	2.064 (1.473, 2.894)	<.0001
Presence of lung metastases	No (reference)	123	80 (43)	—	—
	Yes	101	88 (13)	1.556 (1.148, 2.108)	.0044
Presence of other metastases	No (reference)	74	49 (25)	—	—
	Yes	150	119 (31)	0.931 (0.666, 1.303)	.6783
Metastatic status at 1L initiation	M1c (reference)	145	118 (27)	—	—
	M0	1	1 (0)	13.08 (1.717, 99.64)	.0131
	M1a	19	14 (5)	0.579 (0.332, 1.009)	.054
	M1b	26	20 (6)	0.753 (0.468, 1.213)	.2437
	Mx	33	15 (18)	0.448 (0.261, 0.767)	.0034
adiation prior to 1L initiation	No (reference)	139	95 (44)	—	—
	Yes	85	73 (12)	1.632 (1.196, 2.227)	.002
Surgical resection prior to 1L initiation	No (reference)	69	51 (18)	—	—
	Yes	155	117 (38)	0.812 (0.584, 1.128)	.2147
1L treatment	BRAF/MEKi (reference)	143	122 (21)	—	—
	aPD‐1	81	46 (35)	0.657 (0.467, 0.923)	.0154

Abbreviations: 1L, first‐line; ALT, alanine aminotransferase; aPD‐1, anti‐PD‐1 monotherapies; AST, aspartate aminotransferase; BMI, body mass index; BRAF/MEKi, BRAF/MEK inhibitors; CI, confidence interval; ECOG, Eastern Cooperative Oncology Group; HR, hazard ratio; LDH, lactate dehydrogenase; OS, overall survival; rwPFS, physician‐assessed progression‐free survival.

**TABLE 4:** Multivariable Cox regression models on overall survival and physician‐assessed progression‐free survival from 1L treatment initiation

Covariate	Level	Total	Event (censored)	HR (95%CI)	*P*‐value
OS					
Age at 1L initiation	Per year increase	224	106 (118)	1.044 (1.027, 1.063)	<.0001
Stage at diagnosis	Stage I/II (reference)	21	9 (12)	‐‐	‐‐
	Stage III/IV	167	91 (76)	2.053 (1.025, 4.111)	.0423
	No information	36	6 (30)	0.867 (0.301, 2.497)	.7914
Presence of brain metastases	No (reference)	154	64 (90)	‐‐	‐‐
	Yes	70	42 (28)	2.192 (1.452, 3.309)	.0002
1L treatment	BRAF/MEKi (reference)	143	80 (63)	‐‐	‐‐
	aPD‐1	81	26 (55)	0.602 (0.382, 0.949)	.0287
LDH result	Normal (reference)	93	41 (52)	‐‐	‐‐
	Elevated	53	32 (21)	2.146 (1.327, 3.471)	.0019
	Not documented	78	33 (45)	1.094 (0.683, 1.752)	.7082
rwPFS					
Surgical resection prior to 1L initiation	No (reference)	69	51 (18)	‐‐	‐‐
	Yes	155	117 (38)	0.749 (0.536, 1.047)	.0905
Radiation prior to 1L initiation	No (reference)	139	95 (44)	‐‐	‐‐
	Yes	85	73 (12)	1.793 (1.303, 2.466)	.0003
1L treatment group ‐ patients with rwPFS time < 6 months	BRAF/MEKi (reference)	66	61 (5)	‐‐	‐‐
	aPD‐1	44	35 (9)	1.146 (0.755, 1.738)	.522
1L treatment group ‐ patients with rwPFS time ≥ 6 months	BRAF/MEKi (reference)	77	61 (16)	‐‐	‐‐
	aPD‐1	37	11 (26)	0.228 (0.106, 0.493)	.0002

Abbreviations: 1L, first‐line; aPD‐1, anti‐PD‐1 monotherapies; BRAF/MEKi, BRAF/MEK inhibitors; CI, confidence interval; HR, hazard ratio; LDH, lactate dehydrogenase; OS, overall survival; rwPFS, physician‐assessed progression‐free survival.

Prior radiation therapy (HR = 1.793 [95%CI 1.303‐2.466]; *P* = .0003) was significantly associated with an increased risk of progression or death (Table 4). Among patients without an event within the first 6 months of 1L treatment initiation, receipt of aPD‐1 was associated with a decreased risk of progression or death from 6 months onwards (HR = 0.228 [95%CI 0.106‐0.493]; *P* = .0002). No statistically significant difference between treatment groups was observed in the risk of disease progression or death within 6 months of 1L treatment initiation (HR = 1.146 [95% CI 0.755‐1.738] *P* = .522).

### 3.4 Propensity score analysis

After propensity score matching, 49 matched pairs were selected for analysis. Standardized differences of baseline covariates before and after matching are presented in Table 5 and baseline characteristics of the matched pairs are presented in Table 6. The median OS for those receiving aPD‐1 vs BRAF/MEKi was 27.3 months (95%CI 11.4‐NR) and 13.9 months (95%CI 11.8‐36.5), respectively. The median rwPFS was 5.1 months (95%CI 2.6‐8.1) and 6.8 months (95%CI 5.2‐10.6), respectively. A multivariable Cox regression analysis was performed: older age at 1L initiation (HR = 1.038 per year increase; 95%CI 1.011‐1.067; *P* = .0065) and low albumin (HR = 2.724; 95%CI 1.358‐5.46; *P* = .0048) were statistically associated with an increased risk of death. Among patients who had not died or experienced progression within 10 months of 1L treatment initiation, receipt of aPD‐1 was associated with a statistically lower risk of progression or death from 10 months onwards (HR = 0.252; 95%CI 0.074‐0.856; *P* = .0272). No other factors were significantly associated with death or progression. Accordingly, among patients who experienced death or progression within 10 months of 1L initiation, no statistically significance between treatment groups was observed (HR = 1.308; 95%CI 0.787‐2.174; *P* = .3006).

**TABLE 5:** Standardized differences of baseline covariates before and after propensity score matching

Covariate	Before PS matching ‐ means			After PS matching ‐ means		
	BRAF/MEKi	aPD‐1	Standardized difference^a^	BRAF/MEKi	aPD‐1	Standardized difference
Age at 1L initiation	61.118638	62.893807	0.124	61.397791	62.630218	0.055
BMI at 1L initiation	28.160065	28.091854	0.012	27.77051	27.504839	0.030
Deyo‐adapted Charlson Score	1.1574803	1.3209877	0.103	1.0619455	1.2157635	0.074
Brain metastases	0.3700787	0.2098765	0.359	0.3026492	0.282466	0.044
Lung metastases	0.496063	0.2839506	0.446	0.4578971	0.4458166	0.024
Liver metastases	0.3070866	0.0864198	0.578	0.2198274	0.20815	0.028
Bone metastases	0.2992126	0.2098765	0.206	0.3089958	0.3113862	0.005
Other metastases	0.7322835	0.5555556	0.376	0.6873984	0.639114	0.102
ECOG performance status 0‐1	0.5826772	0.7530864	0.368	0.6058463	0.5896688	0.033
ECOG performance status 2+	0.2204724	0.0493827	0.517	0.1523432	0.1494931	0.008
ECOG performance status not documented	0.1968504	0.1975309	0.002	0.2418106	0.2608381	0.044
Stage at diagnosis III/IV	0.8267717	0.6296296	0.454	0.7199062	0.721749	0.004
Stage at diagnosis I/II	0.0866142	0.0740741	0.046	0.0767331	0.0789891	0.008
Stage at diagnosis not documented	0.0866142	0.2962963	0.553	0.2033607	0.1992619	0.010
Albumin result – low	0.3464567	0.1481481	0.472	0.2714096	0.2513166	0.046
Albumin result – normal	0.503937	0.7901235	0.628	0.6100672	0.632766	0.047
Albumin result ‐ not documented	0.1496063	0.0617284	0.289	0.1185232	0.1159174	0.008
Female	0.3543307	0.382716	0.059	0.3917868	0.3827421	0.019
Practice region ‐ South	0.5669291	0.5432099	0.048	0.5134602	0.5636567	0.101
Practice region ‐ West	0.3149606	0.2716049	0.095	0.2924953	0.2746459	0.040
Practice region ‐ Midwest	0.0708661	0.1111111	0.140	0.1267328	0.0757432	0.170
Practice region ‐ Northeast	0.0472441	0.0740741	0.113	0.0673117	0.0859542	0.070
Prior radiation	0.3700787	0.3703704	0.001	0.3939133	0.4177884	0.049
Prior surgery	0.6614173	0.7530864	0.202	0.7084217	0.711024	0.006
LDH result ‐ normal	0.3700787	0.5061728	0.277	0.4089619	0.3629776	0.095
LDH result ‐ elevated	0.2519685	0.1851852	0.162	0.2306331	0.2646264	0.079
LDH result ‐ not documented	0.3779528	0.308642	0.146	0.360405	0.372396	0.025
History of tobacco exposure	0.488189	0.5185185	0.061	0.5264428	0.5011438	0.051
History of tobacco exposure ‐ not documented	0.015748	0.0246914	0.064	0.0144088	0.0162188	0.015

Abbreviations: 1L, first‐line; aPD‐1, anti‐PD‐1 monotherapies; BMI, body mass index; BRAF/MEKi, BRAF/MEK inhibitors; ECOG, Eastern Cooperative Oncology Group; LDH, lactate dehydrogenase.

^a^ Any standardized difference of more than 0.2 suggests an balanced between the two groups.

**TABLE 6:** Baseline characteristics of propensity score‐matched pairs

Variable	APD‐1 (n = 49)	BRAF/MEKi (n = 49)
Median age at 1L initiation, years (range)	58 (26, 90+)	64 (40, 90+)
Race, n (%)		
White	44 (89.8)	45 (91.8)
Unknown	3 (6.1)	4 (8.2)
Other	2 (4.1)	0 (0.0)
Male sex, n (%)	32 (65.3)	33 (67.3)
Median follow‐up time from 1L initiation, months (range)	11.3 (0.4, 40.5)	11.8 (0.9, 42.3)
ECOG performance status at 1L initiation, n (%)		
0‐1	34 (69.4)	33 (67.3)
2+	3 (6.1)	5 (10.2)
Not documented	12 (24.5)	11 (22.4)
Stage at diagnosis, n (%)		
Stage I/II	5 (10.2)	5 (10.2)
Stage III/IV	36 (73.5)	35 (71.4)
Not documented	8 (16.3)	9 (18.4)
PD‐L1 status, n (%)		
Positive	5 (10.2)	1 (2.0)
Negative	7 (14.3)	3 (6.1)
Not documented	37 (75.5)	45 (91.8)
LDH status at 1L initiation, n (%)		
Normal	22 (44.9)	26 (53.1)
Elevated	8 (16.3)	10 (20.4)
Not documented	19 (38.8)	13 (26.5)
Sites of metastases at 1L initiation, n (%)		
Other	32 (65.3)	28 (57.1)
Lung	19 (38.8)	21 (42.9)
Bone	12 (24.5)	15 (30.6)
Brain	13 (26.5)	14 (28.6)
Liver	7 (14.3)	9 (18.4)
Prior radiation and brain metastases at 1L initiation, n (%)	10 (20.4)	10 (20.4)
Metastatic status at 1L initiation, n (%)		
M0	0 (0.0)	1 (2.0)
M1a	6 (12.2)	5 (10.2)
M1b	7 (14.3)	5 (10.2)
M1c	32 (65.3)	32 (65.3)
Mx	4 (8.2)	6 (12.2)

Abbreviations: 1L, first‐line; aPD‐1, anti‐PD‐1 monotherapies; BRAF/MEKi, BRAF/MEK inhibitors; ECOG, Eastern Cooperative Oncology Group; LDH, lactate dehydrogenase; PD‐L1, programmed death ligand‐1.

## 4 DISCUSSION

In this study, patients with BRAF‐mutant advanced melanoma who received treatment in the USON had demographic and clinical characteristics similar to those reported by similar studies performed in the community oncology setting. Prior research demonstrated that most patients (55.2%) in the US are diagnosed with melanoma before the age of 55, with a higher proportion of white (95.0%) and male (55.2%) patients.^25^ As for BRAF‐mutant patients, Whitman et al (2019), in a retrospective analysis of the Flatiron Health database (n = 454 BRAF‐mutant), reported the median age at 1L treatment initiation to be 63 years, with 92.3% white, 66.7% male, and 83.9% with a 0‐1 ECOG status.^26^ These trends were reflected in our results, which found that among patients with BRAF‐mutant disease who initiated 1L treatment, over half (62.5%) were younger than 65 years, and the majority (89.7%) were white and/or male (62.9%). Additionally, 63.4% had a 0‐1 ECOG status.

In the present study, most patients lacked documentation for PD‐L1 status. PD‐L1 testing is not a requirement for aPD‐1 or BRAF/MEKi.^27^, ^28^ As such, inconsistent use of PD‐L1 testing for treatment selection has been described across National Comprehensive Cancer Network (NCCN) institutions.^3^

The proportions of patients advancing from 1L to 2L and 3L are consistent with similar studies of community‐based care of melanoma. In the real‐world study of patients with BRAF‐mutant advanced melanoma initiating 1L treatment, Luke et al (2019) reported that only 49.8% received 1L treatment, 43.5% received both 1L and 2L, and only 6.7% received 3L.^29^ In another real‐world study of treatment‐naïve patients with BRAF‐mutant advanced melanoma, Whitman et al (2019) found that 57.0% of the BRAF‐mutant cohort received only 1L, 28.0% 1L and 2L, and 15.0% 3L+.^26^ In the present study, 53.1% of the study population received 2L and 17.9%, 3L.

Current NCCN guidelines list nivolumab and pembrolizumab as preferred (Category 1) regimens, with BRAF/MEKi as preferred regimens among patients with BRAF V600‐activating mutations.^3^ Nivolumab/ipilimumab is also recommended for patients willing and able to tolerate the increased toxicity associated with this regimen. The most common 1L treatments observed in this study are in accordance with these guidelines. Like Whitman et al (2019), approximately one‐third of this study population received 1L aPD‐1. Due to the lack of comparative Phase III trials, the NCCN guidelines recommend choosing 1L treatment based on the speed of progression, cancer‐related symptoms, autoimmune disease, or risk status.^3^

The most common 2L treatments in this study, pembrolizumab monotherapy, nivolumab/ipilimumab, dabrafenib/trametinib, and nivolumab monotherapy, are recommended by NCCN guidelines as second‐ and subsequent‐line treatments.^3^ Patients who received 1L aPD‐1 monotherapy most commonly received 2L treatment with a BRAF/MEKi combination therapy; conversely, immunotherapies were the most common 2L treatments among patients who received 1L BRAF/MEKi.

In this study, aPD‐1 was associated with improved outcomes, including OS, rwPFS, DOR, and TTNT, following 1L initiation among patients with BRAF‐mutant advanced melanoma compared to BRAF/MEKi. The lack of statistical significance observed in the propensity score matching results may be due to the relatively small sample size, and these results may nonetheless have clinical relevance. This favorable profile is consistent with published clinical trials, including KEYNOTE‐006 and CheckMate 067.^5^, ^12^

For this study, the median rwPFS was 7.6 months among the aPD‐1 group, which was shorter than the 11.6 months reported in KEYNOTE‐006 for the subset of treatment‐naïve patients who received pembrolizumab.^5^ This difference may be due to underlying differences in this real‐world population compared to the clinical trial, or approach to assessment of progression. In contrast, KEYNOTE‐006 reported a similar 24‐month OS rate, 58.0%, among the subset of patients who received 1L as observed in this study (57.7%). Median OS was not reached among aPD‐1 patients in this study or the subset of 1L BRAF‐mutant patients in KEYNOTE‐006.

Nearly 90% of patients in this study discontinued their 1L treatment, with a higher number of BRAF/MEKi patients discontinuing than those who received aPD‐1. While BRAF/MEKi patients could have had greater data maturity given the FDA approval history, the median follow‐up durations were similar between groups (11.5 months for BRAF/MEKi and 11.3 months for aPD‐1).

The top two reasons for treatment discontinuation were the same for both treatment groups: disease progression (45.2% and 38.1% of aPD‐1 and BRAF/MEKi patients, respectively) and treatment‐related toxicities (11.3% and 14.2% of aPD‐1 and BRAF/MEKi patients, respectively). Disease progression was also the most common treatment discontinuation reason in pooled data from four clinical trials; specifically, 82% of BRAF‐mutant patients discontinued due to progression, with 5% discontinuing due to treatment‐related toxicity.^17^ In KEYNOTE‐001, 42% discontinued due to progressive disease, 25% due to toxicity, 12% due to physician decision, and 5% due to patient withdrawal, with less than 1% lost to follow‐up.^30^ In CheckMate 067, 7.7% discontinued due to treatment‐related adverse events, respectively.^12^

Limited real‐world data have compared aPD‐1 and BRAF/MEKi. Luke et al (2019) retrospectively assessed BRAF‐mutant metastatic melanoma patients treated with immunotherapies (nivolumab/ipilimumab or aPD‐1) or BRAF/MEKi in the community oncology setting. The au­thors report similar durations of therapy between patients who re­ceived dabrafenib/trametinib or aPD‐1 (11.4 vs 12.0 months, respectively).^29^ Moreover, Whitman et al (2019) observed a median OS of 20.7 months for those receiving aPD‐1 and 12.0 months for BRAF/MEKi.^26^ They also observed 1‐year estimated survival rates of 67.5% and 48.9% for the respective treatments.

Several factors may interfere with comparisons across studies. Differences may exist between patient characteristics and eligibility criteria of this study vs other studies, both retrospective observational studies and clinical trials. While the Whitman et al (2019) study included both BRAF‐positive and BRAF wild‐type disease, it did not measure OS for the separate groups.^26^ Also, in Whitman et al (2019), 12.1% had a history of brain metastases compared with 31.3% in the present study. In Luke et al (2019), 11.3% of those receiving aPD‐1 had a history of brain metastases compared with 21.0% in our study, and 9.9% of those treated with targeted therapy had a history of brain metastases compared with 37.1% in the BRAF/MEKi 1L group in our study.^29^ Also, Luke et al (2019) reported the proportions of patients with brain metastases in the BRAF/MEKi and aPD‐1 groups, but did not report a significant difference. More patients in Luke et al (2019) had liver metastases compared with our study (32.4% vs 8.6% in the respective aPD‐1 groups and 46.3% vs 30.8% in the BRAF/MEKi groups). Also, treatment outcomes were measured in different ways across studies and/or reported differently across publications, making comparison difficult. For example, in clinical trials, RECIST 1.1 or immune‐related response is generally used to measure response (eg, CheckMate 067, KEYNOTE‐001, and KEYNOTE‐006), whereas in this study, provider‐assessed response assessments were used.^12^, ^30^, ^31^ Secondly, patients in our study initiated 1L treatment over a 4‐year period, whereas in clinical trials, patients initiate treatment within a much shorter time period.

As a retrospective observational study, underlying patient characteristics may have confounded treatment selection, which would have contributed to the observed clinical outcome differences between patients who received BRAF/MEK treatment and those who received aPD‐1. Attempts were made to control for these differences using multivariable Cox regression analyses and propensity score matching. Based on these models, there was an increased risk of death of about 4% with each yearly increase in age and patients diagnosed with Stage III‐IV disease, brain metastases, and/or elevated LDH having approximately twice the mortality rate. Conversely, receipt of 1L aPD‐1 was associated with a 60% lower risk of death. A similar clinical benefit of aPD‐1 was observed among the propensity score‐matched pairs.

Prior radiation was associated with an 80% increase in risk of progression. Approximately 20% of patients across both cohorts had both prior radiation and brain metastases at initiation of 1L treatment. Given the known association between these variables, inclusion of both in the Cox regression models as separate variables may have influenced the findings. Future studies may consider adjusting for patients with both of these risk factors.

Based on the Cox multivariable regression model, 1L treatment category was not associated with risk of disease progression or death within 6 months of treatment initiation. However, receipt of aPD‐1 was associated with a 23% lower risk of progression than BRAF/MEKi after 6 months of follow‐up. Likewise, among propensity score‐matched pairs, receipt of aPD‐1 was associated with a statistically lower risk of progression or death.

Conclusions about the study results must be drawn in the context of the strengths and limitations of the data source and study design. As a retrospective, observational EHR study, limitations include potential missing or incomplete data. Firstly, data on services provided outside of USON practices would not be available in the USON EHR database. Secondly, EHR data are recorded for clinical care, not for research, which may have resulted in data errors of omission and commission. For example, certain variables of interest to the study, such as PD‐L1 status, were not always available for the entire study population. Thirdly, generalizability to the entire the US population of patients with advanced melanoma may be limited due to the location distribution of USON practices and their use of evidence‐based guidelines.

However, the study had several strengths. First, the study used structured EHR data supplemented by targeted chart review to locate data from unstructured fields within the EHR. These data came from the USON, which is a large network of community‐based oncology practices. Use of the EHR data represents usual care in a large network of community oncology practices. Therefore, these data can be used to report real‐world findings that are more representative of typical patients with melanoma compared with clinical trials with their more restrictive study populations. Second, the propensity score‐matched pairs analysis added robustness to the analysis.

## 5 CONCLUSION

This study provides insight into the real‐world treatment landscape for advanced melanoma and suggests that receipt of 1L aPD‐1 is associated with favorable outcomes among patients with BRAF‐mutant advanced melanoma. While multivariable Cox proportional hazard regression models and propensity score matching were performed to adjust for patient characteristics that may have influenced results, there may be additional underlying factors that were not controlled. Future research should confirm these results through clinical trials and explore factors associated with disease progression in less than 6 months and their relationship with clinical outcomes.

## CONFLICT OF INTEREST

Dr Cowey reports that he provided consulting services to Merck & Co., Inc, Kenilworth, NJ, USA during the conduct of this study. Mr Boyd, Ms Aguilar, and Ms Beeks report that they were employed by McKesson Life Sciences and provided consulting services to Merck & Co., Inc, Kenilworth, NJ, USA during the conduct of this study. Dr Krepler and Ms Scherrer report that they were employed by Merck & Co., Inc, Kenilworth, NJ, USA during the conduct of this study.

## AUTHOR CONTRIBUTIONS

Dr Cowey, Dr Krepler and Ms Scherrer report they contributed to the design of the study, interpretation of results, and critical review of the manuscript. Mr Boyd reports that he contributed to the design of the study, acquisition of the data, analysis of the data, and critical review of the manuscript. Ms Aguilar reports that she contributed to the design of the study, interpretation of the results, and drafting of the manuscript. Ms Beeks reports that she contributed to the acquisition of the data, analysis of the data, and critical review of the manuscript. All authors read and approved the final manuscript.

## Supporting information

Figure S1

## Data Availability

The health data used to support the findings of this study are restricted by the US Oncology Institutional Review Board in order to protect patient privacy. For this reason, data used to support the findings of this study have not been made available.
