# Supplementary material for: An observational study of drug utilization and associated outcomes among adult patients diagnosed with BRAF‐mutant advanced melanoma treated with first‐line anti‐PD‐1 monotherapies or BRAF/MEK inhibitors in a community‐based oncology setting
Source: Cancer Med. 2020 Sep 1;9(21):7863–78. doi: 10.1002/cam4.3312 (PMC7643646; doi:10.1002/cam4.3312)
Supplement: Supplementary file 1 — Figure S1 [file CAM4-9-7863-s001.pdf]

Supplemental Figure 1. Study attrition

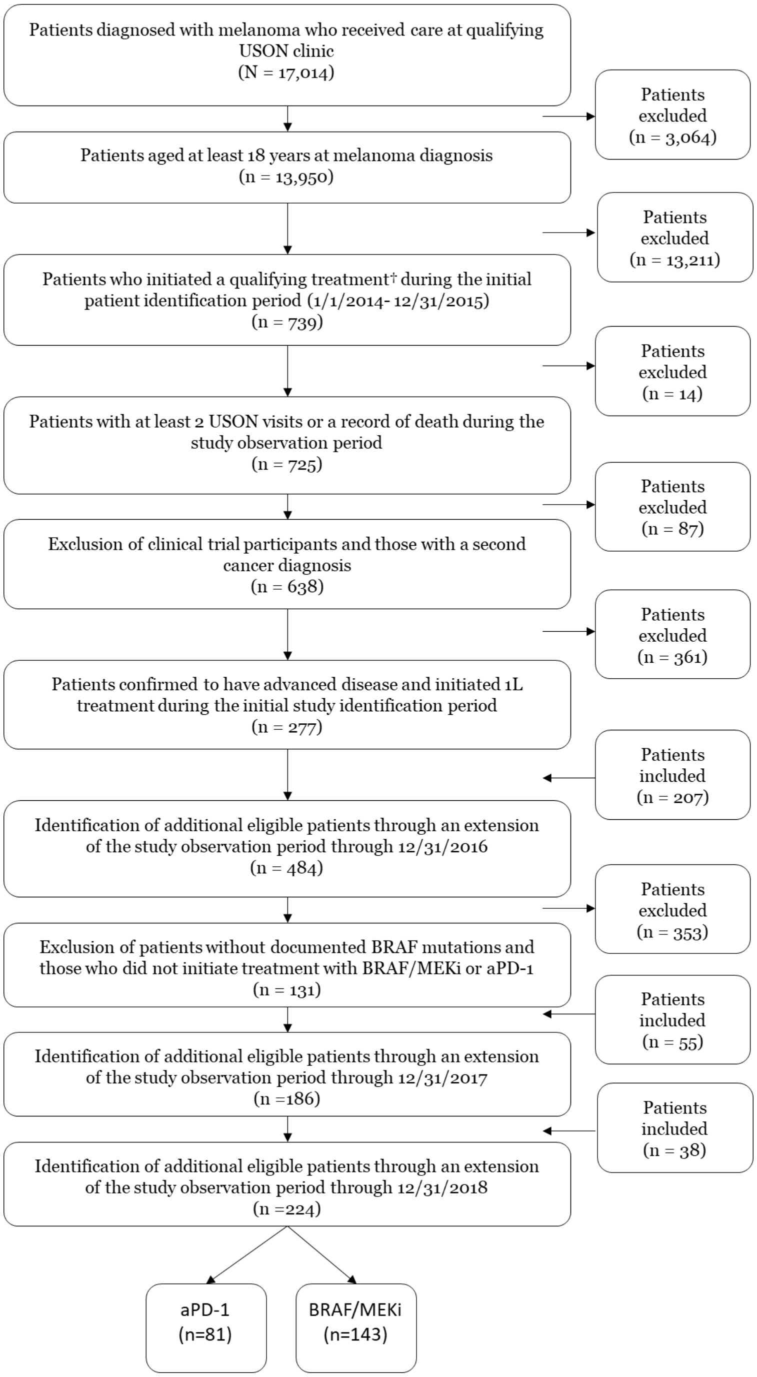

Abbreviations: 1L, first-line treatment; aPD-1, anti-PD1 monotherapies; BRAF/MEKi, BRAF/MEK combination therapy; USON, US Oncology Network

†As the initial study dataset was generated for an expanded study of melanoma treatment patterns, patients included in this count initiated 1L treatment with any anticancer regimen or received pembrolizumab in any line of therapy.
